# Profile of Bile Acid Metabolomics in the Follicular Fluid of PCOS Patients

**DOI:** 10.3390/metabo11120845

**Published:** 2021-12-06

**Authors:** Xiao Yang, Richao Wu, Dan Qi, Linlin Fu, Tian Song, Ying Wang, Yuehong Bian, Yuhua Shi

**Affiliations:** 1 Center for Reproductive Medicine, Cheeloo College of Medicine, Shandong University, Jinan 250012, China; yangxiaosduivf@163.com (X.Y.); Wurichao1996@163.com (R.W.); qidan971215@foxmail.com (D.Q.); fulinlin0119@163.com (L.F.); songtian916@163.com (T.S.); 18663708813@163.com (Y.W.); aa19810603@163.com (Y.B.); 2 Key Laboratory of Reproductive Endocrinology of Ministry of Education, Shandong University, Jinan 250012, China; 3 Shandong Key Laboratory of Reproductive Medicine, Jinan 250012, China; 4 Shandong Provincial Clinical Research Center for Reproductive Health, Jinan 250012, China; 5 National Research Center for Assisted Reproductive Technology and Reproductive Genetics, Shandong University, Jinan 250012, China

**Keywords:** polycystic ovary syndrome, bile acids metabonomic, follicular fluid, ovarian micro-environment

## Abstract

Polycystic ovary syndrome (PCOS) is a complex heterogeneous endocrine disease affected by genetic and environmental factors. In this manuscript, we aimed to describe the composition of bile acid metabolomics in the follicular fluid (FF) of PCOS. The FF was collected from 31 control patients and 35 PCOS patients diagnosed according to the Rotterdam diagnostic criteria. The Bile Acid Assay Kit and ultra-performance liquid chromatography/tandem mass spectrometry (UPLC-MS/MS) were used in this study to detect the total bile acid and 24 bile acid metabolites. Glycocholic acid (GC3A), taurocholic acid (TCA), glycochenodeoxycholic acid (GCDCA), and chenodeoxycholic acid-3-β-d-glucuronide (CDCA-3Gln) were elevated in the PCOS group. GCDCA was positively correlated with the serum follicle-stimulating hormone (FSH) (r = 0.3787, *p* = 0.0017) and luteinizing hormone (LH) (r = 0.2670, *p* = 0.0302). The level of CDCA-3Gln also rose with the increase in antral follicle counts (AFC) (r = 0.3247, *p* = 0.0078). Compared with the control group, the primary bile acids (*p* = 0.0207) and conjugated bile acids (*p* = 0.0283) were elevated in PCOS. For the first time, our study described the changes in bile acid metabolomics in the FF of PCOS patients, suggesting that bile acids may play an important role in the pathogenesis of PCOS.

## 1. Introduction

Polycystic ovary syndrome (PCOS) is a complex endocrine and metabolic disorder, and is one of the main causes of anovulatory infertility. According to different diagnostic criteria, the prevalence of PCOS in women of childbearing age is approximately 6~20% [1]. In addition to suffering from infertility, women with PCOS often experience metabolic disorders, and they have an increased risk of type 2 diabetes and cardiovascular disease in the long term [2,3,4,5]. Therefore, in order to reduce the psychological burden and social pressure of PCOS patients, many researchers have devoted themselves to revealing the pathogenesis of PCOS and seeking better diagnosis and treatment strategies for patients.

Follicular fluid (FF) is composed of plasma components, follicle secretions, and secretory components of other ovarian cells (ovarian granulosa cells and theca cells), which regulates the micro-environment of the ovary and affects the occurrence and development of oocytes. An important pathophysiological change in PCOS is the increase in the number of antral follicles, causing sparse ovulation, which usually manifests as irregular menstruation in the clinic. The pathogenesis of PCOS is usually accompanied by the disorders of proliferation and apoptosis in ovarian granulosa cells [6,7]. Compared with the FF of patients without PCOS, the FF of PCOS patients had higher androgen levels and lower estrogen levels [8]. The alteration of the follicular micro-environment could affect the proliferation of granulosa cells and the arrest of oocytes [9].

Bile acid is a constituent of bile synthesized by the liver and is related to glucose and lipid metabolism. Primary bile acid is biosynthesized in the liver and then decomposed by intestinal flora in the intestine into secondary bile acids. According to whether bile acids are combined with glycine or taurine, they are divided into conjugated bile acids and unconjugated bile acids. In addition to regulating their own synthesis, bile acids have an endocrine function [10]. In recent years, many studies have been devoted to revealing the possible role of bile acids in the pathogenesis of PCOS [11]. In serum, there is a positive correlation with increased circulating conjugated primary bile acids and hyperandrogenemia in PCOS patients [12]. However, there is still no research on bile acid metabolites in the ovary micro-environment of PCOS.

In this manuscript, we reported 24 bile acid metabolites detected by ultra-performance liquid chromatography/tandem mass spectrometry (UPLC-MS/MS) in the FF of PCOS patients and performed quantitative analysis. For the first time, we focused on the bile acid metabolomics of FF in PCOS patients, revealed the differences in bile acid metabolites in the FF of PCOS patients, and suggested that they might play a role in the ovarian micro-environment, in order to provide new insight for the pathogenesis and the clinical treatment of PCOS.

## 2. Results

### 2.1. Characteristics of the Participants

The clinical characteristics of all participants (31 control patients and 35 patients with PCOS) involved in this study were summarized in Table 1. As shown in Table 1, there were no significant differences in aspartate transferase (AST), alanine transferase (ALT), gamma-glutamyl transferase (GGT), triglycerides (TG) or blood glucose levels between control patients and PCOS patients, which excluded the influence of liver function on the state of bile acids in FF. The differences in follicle-stimulating hormone (FSH) levels, body mass index (BMI) and the age between the two groups were also not statistically significant. Statistically significant differences in antral follicle counts (AFC) and the serum levels of anti-Mullerian hormone (AMH), luteinizing hormone (LH), testosterone (T) existed between the two groups. These differences were consistent with the clinical characteristics of PCOS patients.

### 2.2. Differences of Total Bile Acids in Follicular Fluid

**Figure 1:**
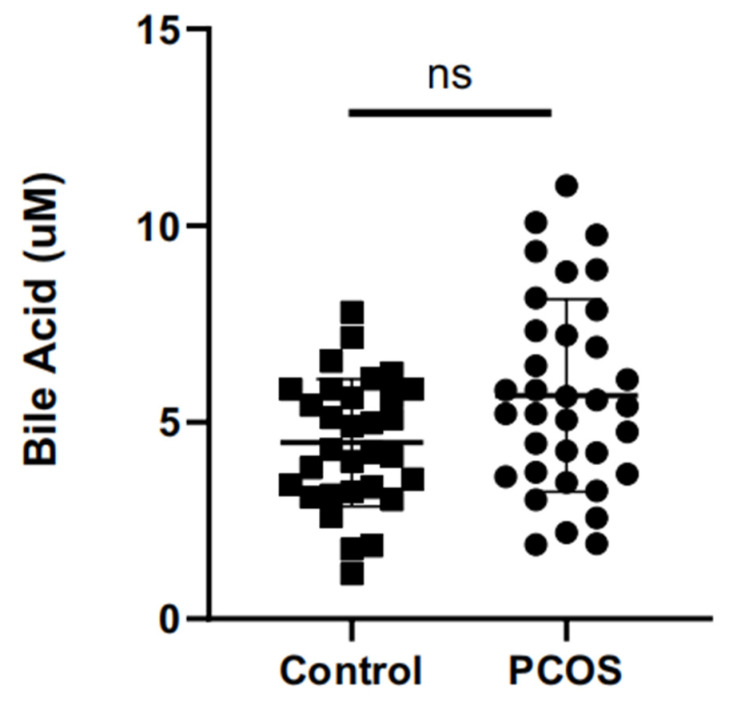
The differences in total bile acids in follicular fluid (FF). ns = not statistically significant.

Compared with the control group, the level of total bile acid showed an increasing trend in FF from the PCOS group, but there was no statistically significant difference (*p* = 0.0640) (Figure 1).

### 2.3. Expression Profile of Bile Acid Metabolites

**Figure 2:**
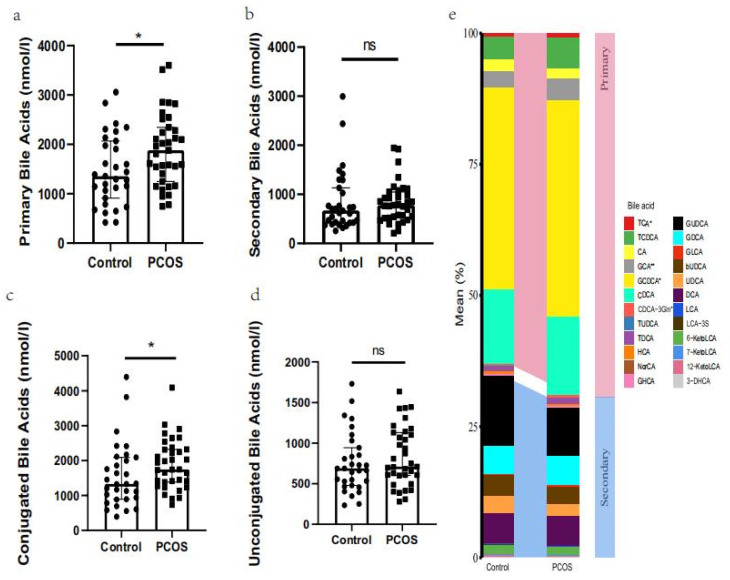
(**a**) Differences in primary bile acids. (**b**) Differences in secondary bile acids. (**c**) Differences in conjugated bile acids. (**d**) Differences in unconjugated bile acids between the control group and the PCOS group (**e**) Expression profile of bile acid metabolites in follicular fluid (FF). * *p* < 0.05, **: Statistical significance; ns = not statistically significant.

In this study, a total of 24 bile acid metabolites were identified by UPLC-MS/MS (Figure 2). Among them, four different bile acid metabolites were detected as being elevated in the PCOS group: GCA (*p* = 0.0088), TCA (*p* = 0.0302), GCDCA (*p* = 0.0380), and CDCA-3Gln (*p* = 0.0489) (Table 2). Furthermore, after further analysis of the bile acid metabolite detection results, it was found that primary bile acids increased significantly in the PCOS group (*p* = 0.0207). However, there was no significant difference in the levels of secondary bile acids in FF between the two groups (*p* = 0.3062). Meanwhile, conjugated bile acids were elevated in PCOS FF (*p* = 0.0283). However, the concentration of unconjugated bile acids was similar between the two groups (*p* = 0.3778).

### 2.4. Correlation between Bile Acid Metabolites and Clinical Characteristics

After analyzing the correlation between the different quantitative results of bile acid metabolites in FF and the clinical characteristics of the patient, it was found that GCDCA was positively correlated with the serum FSH (r = 0.3787, *p* = 0.0017) and LH (r = 0.2670, *p* = 0.0302). The level of CDCA-3Gln also rose with the increase in AFC (r = 0.3247, *p* = 0.0078). There was no statistical correlation between the analysis of other bile acid metabolites and clinical indicators (Table 3).

## 3. Discussion

In this study, we found that the total bile acid in the FF of PCOS patients was not statistically different from that of the control group, but four different bile acid metabolites were screened out as biomarkers. GCA, TCA, GCDCA and CDCA-3Gln were elevated in the FF of PCOS. Compared with the control group, the primary bile acids in the FF of the PCOS group were significantly increased, which was consistent with previous reports on the PCOS serum bile acid profile [12]. GCA, TCA and GCDCA are conjugated bile acids. Additionally, conjugated bile acids were elevated in the PCOS group significantly. After analyzing the correlation between the different bile acid metabolites and the clinical characteristics of the included patients, it was found that GCDCA was positively correlated with the levels of FSH and LH in serum. It is well known that the LH signal was closely related to the occurrence of ovulation [13]. This result implies that the elevated of GCDCA in FF is related to the ovulation disorder in PCOS. Meanwhile, the level of CDCA-3Gln was positively correlated with AFC, which demonstrated that CDCA-3Gln might be related to the ovarian reserve. At the same time, the status of the insulin resistance was assessed by QUICKI. QUICKI did not change with increase in the levels of the four different bile acid metabolites, which suggests that there was no relationship between the insulin resistance and the level of bile acid metabolites in FF. The present results suggest that certain types of bile acid metabolite may affect the ovarian micro-environment of PCOS patients, which may be related to the growth and development of follicular and ovulation disorders.

As for the four different bile acid metabolites detected, in the China Cardiometabolic Disease and Cancer Cohort (4C) Study, it was found that plasma GCA, TCA, and GCDCA were all related to the increased risk of type 2 diabetes mellitus (T2DM) [14]. In the study of hepatocellular carcinoma (HCC), GCA has been identified as a sensitive biomarker which was elevated in the serum and urine of HCC [15,16]. GCDCA induces stemness through the STAT3 pathway, thereby promoting the chemoresistance of HCC, it could become a potential target of HCC chemotherapy [17]. TCA promoted the cellular cellularity of retinal pigment epithelial (RPE) cells and reduced choroidal endothelial cell migration and tube formation caused by vascular endothelial growth factor (VEGF) [18].

In previous research, the presence of bile acid metabolites was found in the ovaries of rats [19]. A report described that all aspects of the bile acid synthesis pathway were present in follicles, and proposed that bile acid is produced by granulosa cells [20]. The level of bile acid in human FF was almost twice that of serum, and an increase in the UDCA derivative level was found in FF, resulting in high-quality embryos [21]. Then, the study of Nagy R. A. et al. suggested that the regulation of bile acid transport from blood to FF may have potential effects on the reproductive system [22]. Bile acids are cytotoxic, and it has been proven that excessive bile acids can impair ovarian function by damaging mitochondrial function and inducing oxidative stress [23]. Differences in bile acid metabolites were found between healthy follicles and atretic follicles of Bama Xiang pigs, suggesting that there may be a disorder of bile acid metabolism in porcine atresia follicles [24].

Ovarian granulosa cells could regulate the growth and development of follicles. The presence of farnesoid X receptor (FXR), which serves as the natural receptor of bile acid, has been confirmed in ovarian granulosa cells, and it may play a role in regulating the function of granular cells, thereby affecting ovarian function [25]. As an essential part of bile acid metabolism, liver receptor homolog 1 (LRH-1) also has a role in gonadal development and ovulation [26,27]. In our previous study, we discovered high LRH-1 expression in the ovarian granulosa cells of PCOS patients [28]. All this evidence indicates that bile acid metabolites are likely to play a role in follicular development and ovarian function in PCOS. Researchers have found that patients with PCOS have an altered intestinal bile acid metabolism [11]. In the research into serum metabolomics mass spectrometry analysis of PCOS patients, GCA was selected as a biomarker, suggesting that bile acid metabolism may play a role in PCOS [29]. We screened GCA among the differential bile acid metabolites in FF, which was confirmed with the results of the serum.

Previous research only focused on the circulating changes in bile acid in PCOS. In our research, we revealed the differences in bile acid metabolites in the FF of PCOS patients. This implied that bile acid metabolites might affect the regulation of the function of ovarian cells and promote the progression of ovulation disorders in PCOS patients.

## 4. Materials and Methods

### 4.1. Participants

In this study, PCOS was diagnosed according to the Rotterdam criteria (Rotterdam, 2004). Based on the Rotterdam criteria, the diagnosis of PCOS requires at least two of the following three points: oligo- and/or anovulation; clinical and/or biochemical evidence of hyperandrogenism; and ovary polycystic morphology under ultrasound. Other diseases that may cause hyperandrogenism and ovulation disorders need to be excluded, such as Cushing’s syndrome, congenital adrenal hyperplasia, and androgen-secreting tumors. The females in control group were those who sought fertility treatment due to oviduct factors or male factors, and they all had regular menstrual cycles. The androgen factor was excluded in the control group. On days 2–5 of the menstrual cycle, with the basal endocrine state, control patients’ serum FSH < 10 IU/L and AMH > 1.5 ng/mL. On the day of using human chorionic gonadotropin (hCG), the follicle count was greater than 5 and less than 15, to ensure the ovarian reactivity in the control group. The study included 35 patients with PCOS and 31 control patients. All participants were younger than 40 years old and they underwent intracytoplasmic sperm injection (ICSI) or in vitro fertilization-embryo transfer (IVF-ET) treatment in the Reproductive Hospital of Shandong University from July 2020 to November 2020. With the guidance of experienced clinicians, a standard long agonist protocol or antagonist protocol was chosen as the ovulation-stimulation regimen. All patients excluded the history of ovarian surgery, hyperprolactinemia, and thyroid disease. According to the guidance of a previous report, the quantitative insulin sensitivity check index (QUICKI) = 1/[log(fasting insulin) + log(fasting plasma glucose)] was calculated to assess the status of insulin resistance [30]. To eliminate the influence of the liver, all patients were confirmed has having normal liver function. The Ethics Committee of the Affiliated Reproductive Hospital of Shandong University approved this study.

### 4.2. The Collection of FF

After observing proper follicle development through transvaginal ultrasound, clinicians instructed the use of hCG to induce ovulation. After 36 h of hCG use, during the retrieving of the oocytes, follicular fluid was collected in a sterile centrifuge tube. As quickly as possible, the fresh FF was transported to the laboratory, and centrifuged at 2000 rcf for 10 min. Then, 1 mL of the supernatant of FF was collected and stored at −80 °C.

### 4.3. The Detection of Total Bile Acid

Before detecting FF with the kit, samples were taken from −80 °C and melted on ice in order to maintain more stable characteristics. The Bile Acid Assay Kit (MAK309) was employed to determine the total bile acid in FF based on the protocol provided by the manufacturer.

### 4.4. FF Bile Acid Profile Assessment

Metabo-Profile Inc. (Shanghai, China) performed the comprehensive profiling and quantitation of the bile acid metabolites using previously published methods [31,32]. The bile acid detection kit-BAP Ultra (Metabo-Profile, Shanghai, China) was used to analyze the bile acid profile in the research. All FF samples were qualified after quality control. Based on UPLC-MS/MS, a total of 24 bile acid metabolites listed in Table 4 were quantified. Primary or secondary bile acids, conjugated or unconjugated bile acids are calculated according to the classification.

### 4.5. Statistical Analysis

Data analysis was performed using SPSS 26.0 (SPSS, Chicago, IL, USA) and GraphPad Prism 8.0 (GraphPad Software, San Diego, CA, USA). A normality test of continuous variables was performed by using Kolmogorov–Smirnov test. Normally distributed variables were analyzed using Student’s t-test to determine statistical significance and displayed in the form of mean ± standard deviation (SD). Non-normally distributed data are displayed in the form of median and quartile, compared using a nonparametric test. The linear association was assessed by using Pearson’s correlational analysis. A *p* value < 0.05 was considered statistically significant.

## 5. Conclusions

For the first time, our study explored the bile acid metabolomics in the FF of patients with PCOS, highlighting the impact of bile acid metabolite changes in the ovarian micro-environment. However, the individual differences of clinical samples cannot be ignored, and so a further study with an expanded sample is still needed to reveal the role of bile acid metabolism in the ovarian function of PCOS patients.

## Figures and Tables

**Table 1:** Clinical characteristics between polycystic ovary syndrome (PCOS) group and control group.

	Control (*n* = 31)	PCOS (*n* = 35)	*p* Value
Age (years)	31.03 ± 3.95	29.23 ± 3.84	0.064
AFC (counts)	14.77 ± 5.61	27.97 ± 11.02	*p* < 0.001
AMH (ng/mL)	3.50 ± 1.60	8.40 ± 4.48	*p* < 0.001
LH (IU/L)	5.98 ± 1.95	11.73 ± 6.89	*p* < 0.001
FSH (IU/L)	6.67 ± 1.38	6.25 ± 1.63	0.267
T (ng/dL)	20.76 ± 9.99	38.13 ± 22.12	*p* < 0.001
BMI (kg/m^2^)	23.14 ± 2.78	24.29 ± 3.00	0.113
Glucose (mmol/L)	5.26 ± 0.43	5.17 ± 0.34	0.345
AST (U/L)	16.96 ± 3.75	17.58 ± 3.20	0.470
ALT (U/L)	13.64 ± 5.29	16.45 ± 7.85	0.096
GGT (U/L)	13.20 ± 4.36	16.06 ± 8.05	0.083
TG (mmol/L)	0.92 ± 0.39	0.98 ± 0.43	0.497
QUICKI	0.56 ± 0.09	0.51 ± 0.11	0.112

AFC, antral follicle counts; AMH, anti-Mullerian hormone; LH, luteinizing hormone; FSH, follicle-stimulating hormone; T, testosterone; BMI, body mass index; AST, aspartate transferase; ALT, alanine transferase; GGT, gamma-glutamyl transferase; TG, triglycerides; QUICKI = 1/[log(fasting plasma insulin) + log(fasting plasma glucose).

**Table 2:** Expression profile of bile acid metabolites in follicular fluid between the two groups.

Bile Acid Metabolites (nmol/L)	Control (*n* = 31)	PCOS (*n* = 35)	*p* Value
GCA ^#^	69.3455 (35.0402–108.194)	93.0293 (60.9071–139.1637)	0.0088
TCA ^#^	8.9051 (6.1289–18.8495)	20.0173 (10.2183–32.6538)	0.0302
GCDCA ^#^	813.247 (567.252–1181.14)	1045.532 (813.6052–1415.405)	0.0380
CDCA-3Gln ^#^	2.0029 (1.4314–4.7204)	4.5151 (1.8605–16.4768)	0.0489
TUDCA	1.1045 (0.4843–3.7344)	1.7891 (0.4667–5.5518)	0.7018
TCDCA	101.073 (58.0077–132.758)	131.5096 (87.4559–197.0543)	0.0586
TDCA	17.21 (6.3327–36.5325)	19.6411 (4.5489–42.3435)	0.6570
HCA	6.5258 (4.3054–11.0712)	7.5380 (4.2251–12.5456)	0.8183
CA	39.3982 (27.9622–56.5257)	41.8243 (29.0361–67.7721)	0.5742
NorCA	1.3382 (0.8645–2.2769)	1.5677 (1.0934–2.4134)	0.3376
GHCA	6.3761 (3.6203–11.9017)	7.7032 (5.1268–12.2494)	0.4282
GUDCA	137.575 (76.6138–343.196)	181.9499 (77.9537–314.5836)	0.9086
GDCA	88.5226 (54.237–145.261)	92.7559 (36.1559–218.6125)	0.5484
GLCA	3.9102 (0.8078–9.2406)	3.3065 (0.6672–13.6760)	0.7278
bUDCA	50.0141 (30.1774–96.4139)	48.4582 (24.3200–105.7436)	0.8985
UDCA	40.5876 (24.5589–65.4812)	40.5971 (19.3277–75.6631)	0.8382
CDCA	352.781 (177.59–457.672)	375.8702 (218.3674–594.6697)	0.3123
DCA	115.298 (55.4131–182.886)	112.2262 (34.3780–209.7214)	0.7497
LCA	2.7428 (1.2261–3.8918)	1.9536 (0.8700–4.8796)	0.5399
LCA-3S	1.5409 (0.3816–2.4319)	0.9994 (0.1386–3.2181)	0.6923
6-KetoLCA	39.4591 (30.957–53.853)	35.8543 (27.1566–47.3019)	0.3311
7-KetoLCA	4.1440 (2.8514–7.435)	3.8017 (2.1842–6.8645)	0.3376
12-KetoLCA	3.1705 (1.1653–6.6363)	1.9183 (0.5242–6.5477)	0.7208
3-DHCA	1.8208 (1.3506–3.4268)	2.1905 (1.4482–3.4760)	0.7400

^#^ Statistical significance.

**Table 3:** The correlation between bile acid metabolites and clinical characteristics.

Bile Acid	Clinical Characteristic	Pearson’s r	*p* Value
TCA	AMH	0.0922	0.4617
	FSH	0.0400	0.7499
	LH	0.0898	0.4735
	E2	0.0286	0.8197
	T	0.2348	0.0577
	AFC	0.1158	0.3546
	QUICKI	0.0050	0.6166
GCA	AMH	0.1590	0.2023
	FSH	0.2027	0.1026
	LH	0.1123	0.3695
	E2	0.0475	0.7051
	T	0.2000	0.1074
	AFC	0.1408	0.2594
	QUICKI	0.0105	0.4649
GCDCA	AMH	0.1424	0.2514
	FSH ^#^	0.3787	0.0017
	LH ^#^	0.2670	0.0302
	E2	0.1129	0.3669
	T	−0.0001	0.9991
	AFC	0.1481	0.2353
	QUICKI	0.0132	0.4125
CDCA-3Gln	AMH	0.2078	0.0940
	FSH	−0.1059	0.3976
	LH	0.0869	0.4878
	E2	0.0170	0.8921
	T	0.1655	0.1841
	AFC ^#^	0.3247	0.0078
	QUICKI	0.0021	0.7466

^#^ Statistical significance; quantitative insulin sensitivity check index (QUICKI).

**Table 4:** Abbreviations of bile acid metabolomics.

Abbreviation	Full Name	Classification (Primary/Secondary)	Classification (Conjugated/Unconjugated)
TCA	Taurocholic acid	Primary	Conjugated
TUDCA	Tauroursodeoxycholic acid	Secondary	Conjugated
TCDCA	Taurochenodeoxycholic acid	Primary	Conjugated
TDCA	Taurodeoxycholic acid	Secondary	Conjugated
HCA	Hyocholic acid	Secondary	Unconjugated
CA	Cholic acid	Primary	Unconjugated
NorCA	Norcholic acid	Secondary	Unconjugated
GHCA	Glycohyocholate	Secondary	Conjugated
GCA	Glycocholic acid	Primary	Conjugated
GUDCA	Glycoursodeoxycholic acid	Secondary	Conjugated
GCDCA	Glycochenodeoxycholic acid	Primary	Conjugated
GDCA	Glycodeoxycholic acid	Secondary	Conjugated
GLCA	Glycolithocholate	Secondary	Conjugated
bUDCA	Isoursodeoxycholic acid	Secondary	Unconjugated
UDCA	Ursodeoxycholic acid	Secondary	Conjugated
CDCA	Chenodeoxycholic acid	Primary	Unconjugated
DCA	Deoxycholic acid	Secondary	Unconjugated
LCA	Lithocholic acid	Secondary	Unconjugated
LCA_3S	Lithocholic acid 3 sulfate	Secondary	Conjugated
6_ketoLCA	6-ketolithocholic acid	Secondary	Unconjugated
7_ketoLCA	7-ketolithocholic acid	Secondary	Unconjugated
12_ketoLCA	12-ketolithocholic acid	Secondary	Unconjugated
3_DHCA	3-oxocholic acid	Secondary	Unconjugated
CDCA_3Gln	Chenodeoxycholic acid-3-β-d-glucuronide	Primary	Conjugated

## Data Availability

The data and materials during this study are available from the corresponding author on reasonable requests. The data are not publicly available due to data protection regulation law.
